# A 12-week application-based group conversation intervention on cognitive health and psychosocial well-being among older adults during the COVID-19 pandemic: a randomized controlled trial

**DOI:** 10.1186/s12877-025-06444-0

**Published:** 2025-10-14

**Authors:** Kumi Watanabe Miura, Takuya Sekiguchi, Seiki Tokunaga, Hikaru Sugimoto, Taishiro Kishimoto, Takashi Kudo, Mihoko Otake-Matsuura

**Affiliations:** 1 Japan Society for the Promotion of Science (JSPS), Tokyo, Japan; 2 RIKEN Center for Advanced Intelligence Project, Tokyo, Japan; 3 Department of Neuropsychiatry, School of Medicine, Keio University, Tokyo, Japan; 4 Department of Psychiatry, Graduate School of Medicine, Osaka University, Osaka, Japan

**Keywords:** Cognitive health, Social isolation, Loneliness, Mobile applications, Communication technology, Randomized controlled trial

## Abstract

**Background:**

Following empirical evidence suggesting a strong connection between social activities and longevity/well-being, this topic has now entered an intervention phase, which evaluates the effectiveness of social activity interventions and accumulates practical knowledge. Among these interventions, conversation—as a core component of social activity—has emerged as a promising target for cognitive, social, and psychological health.

**Objective:**

This study examined the effects of “Photo-Integrated Conversation Moderated by Application” (PICMOA), an application-based remote conversational intervention, on the cognitive function and psychological and social well-being, as reflected by selected indicators, among community-dwelling older adults with subjective cognitive concerns in Japan.

**Methods:**

The PICMOA trial is a randomized controlled trial introducing an open-label, two parallel group trial design with a 1:1 allocation. In this trial, the effects of the PICMOA intervention, a weekly application-based group conversation, were compared with weekly health education videos. The participants included community dwellers aged 65 years and older with subjective cognitive concerns. The main outcomes included cognitive function assessed through the Telephone Interview for Cognitive Status in Japanese, verbal fluency test, and Digit Span Forward and Backward tests. The secondary outcomes included indicators related to psychological and social well-being, assessed using the Multidimensional Scale of Perceived Social Support, the UCLA Loneliness Scale-10 item version, the 5-item World Health Organization Well-Being Index, and the Health Utilities Index Mark 3.

**Results:**

In total, 81 participants were randomized and divided into two groups (intervention: *n* = 41; control: *n* = 40). A total of 75 participants who completed the intervention and evaluations were analyzed (intervention: *n* = 35; control: *n* = 40). There were no significant improvements associated with cognitive function and psychological metrics in the intervention group compared with the control group during the 12-week intervention. However, the ancillary analysis showed significant decline in the categorical fluency performance for those who were unfamiliar with smartphones at baseline (b = -5.47, SE = 2.04, *P* = 0.009), suggesting a moderating effect of participants’ familiarity with smartphones at baseline.

**Conclusions:**

This trial showed no significant improvements in cognitive and psychological outcomes after the PICMOA intervention. However, the findings raise important considerations regarding participants’ familiarity with digital devices and intervention setting. Further research is needed to accumulate evidence on the duration and intensity of intervention and individual support for improving digital literacy.

**Clinical trial number:**

UMIN000047247 (http://www.umin.ac.jp/ctr/), registered on March 22, 2022.

**Supplementary Information:**

The online version contains supplementary material available at 10.1186/s12877-025-06444-0.

## Introduction

Social activity may play a key role in preventing dementia and maintaining well-being in later life. In light of the vast empirical evidence on the relationship between social activity and longevity/well-being outcomes [1–5], this topic has now entered the intervention phase to evaluate the effectiveness of social activity and accumulate practical knowledge through intervention studies.

Conversation—as a core communicative component of social activity—is driven by diverse cognitive functions, including attention, working memory, inhibition, processing speed, and social cognition [6]. These functions are essential for maintaining focus, managing issues, interpreting others’ intentions and feelings, and regulating inappropriate behavior. Additionally, linguistic ability, which is the fundamental ability to have conversations, is closely related to cognitive function. Individuals with mild cognitive impairments (MCI) and those in the initial phases of dementia frequently experience language processing impairments (for a review, see [7]). Linguistic features are also alternated during cognitive decline. A study reported that patients with mild to moderate Alzheimer’s disease exhibited specific linguistic features, such as increased word-finding delays, semantic paraphasia, vacuous and indefinite phases, as well as fewer error corrections and responses to word-finding delays, and fewer long complex phases, as compared to healthy controls [8]. Another study reported a more rapid decline in speech fluency and semantic content of spoken words among those with early MCI as compared to those who are cognitively stable [9]. Furthermore, linguistic features, including speech volume, tense, and vocabulary, have been used as screening measures for cognitive deficits such as MCI and dementia [10–12].

Considering the close association between conversation and cognitive function, it was hypothesized that conversation-based interventions might be beneficial in stimulating cognitive function. Accordingly, Photo-Integrated Conversation Moderated by Robots (PICMOR), a structured group conversation with a robot that facilitates conversation, was developed. Previous randomized controlled trials (RCTs) consistently found specific positive effects of PICMOR on verbal fluency [13, 14], which is closely related to executive functioning and linguistic ability. A 12-week RCT utilizing weekly PICMOR intervention improved phonemic fluency compared with the weekly health education control [13]. Studies utilizing magnetic resonance imaging also found significant differences in brain status at post-intervention, such as prefrontal volume [15], white matter fibers in the frontal region [16], and functional connectivity with the frontal area [17], which are involved in verbal fluency. Additionally, another RCT introducing biological indicators, including blood neurofilament light chain (NfL), was found to have a positive intervention effect on semantic fluency in the group with lower levels of NfL suggesting better cognitive function [14].

Although PICMOR has been conducted through face-to-face interventions, there is growing interest in remote interventions or telemedicine interventions due to innovation in information communication technology and social circumstances (e.g. COVID-19). Remote interventions enable practitioners to approach populations that cannot be reached conventionally, resulting in greater accessibility than was previously only possible with face-to-face interventions. With the benefit of remote interventions, a smartphone application version of PICMOR, “Photo-Integrated Conversation Moderated by Application” (PICMOA), was developed [18].

This study evaluated the effects of a PICMOA intervention on cognitive functioning among Japanese older adults at a risk of cognitive decline. In line with previous RCT studies [13, 14, 19], we hypothesized that the PICMOA intervention, which is an application-based conversational intervention, would be beneficial for maintaining cognitive function through cognitive stimulation during conversational interactions. In addition, the effect on psychological and social well-being, as reflected by selected indicators, was considered based on substantial evidence linking social isolation—a lack of conversational or interactive engagement—with well-being [5, 20]; we hypothesized that the PICMOA intervention may mitigate states related to social and psychological well-being.

## Methods

### Trial design

The PICMOA trial introduced an open-label and two parallel group trial design with a 1:1 allocation. The detailed protocol for the PICMOA trial can be found in the protocol paper [18]. There were no diversions from or amendments to the published protocols.

### Blinding

This study used an open label design because the nature of the intervention meant it was not feasible to blind participants to their assigned condition. The assessors were blinded to the allocation to mitigate potential assessment biases.

### Randomization

Stratified randomizations were conducted utilizing a block design with a 1:1 allocation to either the intervention or control group. We stratified participants based on their gender and subsequently arranged them in accordance with their TICS-J total scores. Subsequently, block randomization with a block size of two was conducted, allocating the groups based on computer-generated random numbers. T. S. was responsible for the allocation process and possessed only the participants’ ID, TICS-J score, and gender during the randomization phase. The entire randomization process was carried out using the statistical software R.

### Participants

**Fig. 1:**
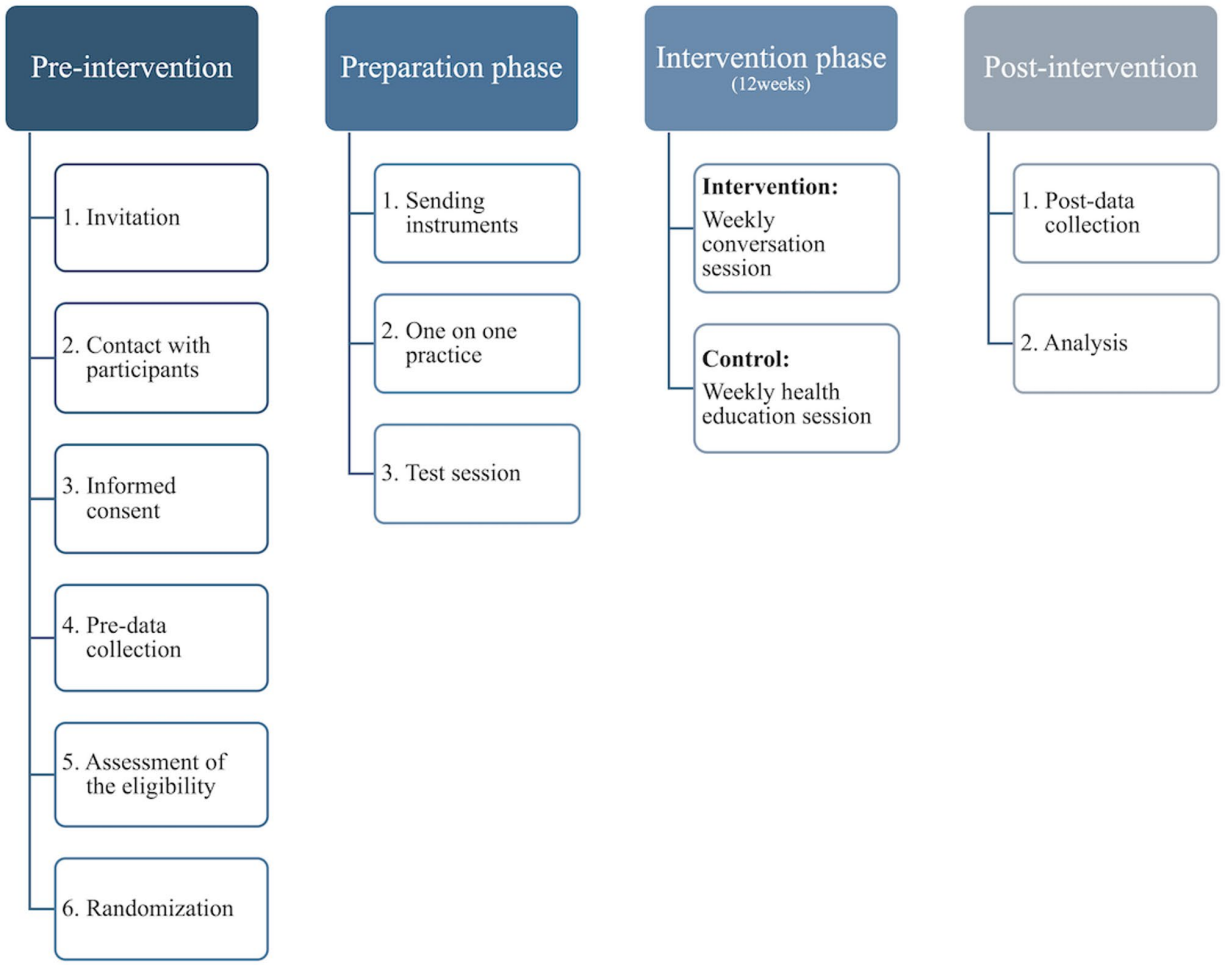
Participant flow diagram

Figure 1 illustrates the CONSORT diagram, which describes participant flow. The participants in the PICMOA trial included community dwellers of Wako city aged 65 years and older who reported subjective cognitive concerns but have no clinical cognitive impairment. Referring to previous RCT studies [21, 22], subjective cognitive concerns were screened using the cognitive functioning domain in the Kihon checklist (KCL) [23]. The KCL is a self-report screening tool recommended by Japan’s Ministry of Health, Labour and Welfare. It has been widely used by municipal governments as a part of public health policy to determine high-risk populations and invite public health interventions. This scale reports predictive validation for the future onset of long-term care needs and mortality [24]. Although individuals with MCI or dementia are also important targets for intervention, we intentionally focused on those in the preventive stage—specifically, individuals with subjective cognitive concerns who are at risk of future cognitive deterioration. By targeting this group, this study aimed to support early prevention efforts aligned with Japan’s public health policy, which emphasizes early detection using KCL and intervention before serious impairment occurs.

The inclusion criteria for this study were as follows: Participants had subjective cognitive concerns screened by the cognitive functioning domain of the KCL; had no clinical MCI or dementia, as indicated by a total score greater than or equal to 33 on the Interview for Cognitive Status in Japanese (TICS-J) [25]; were available to provide written informed consent; and were able to undergo the required assessments and intervention sessions on the specified dates. Conversely, participants were excluded if they had a history of previous medical conditions or medications that could significantly affect the central nervous system (e.g., neurological impairment, stroke, serious head injury, and serious complicating disorders) and certification as “care needs levels” or “support need levels” by the public long-term care insurance system.

**Fig. 2:**
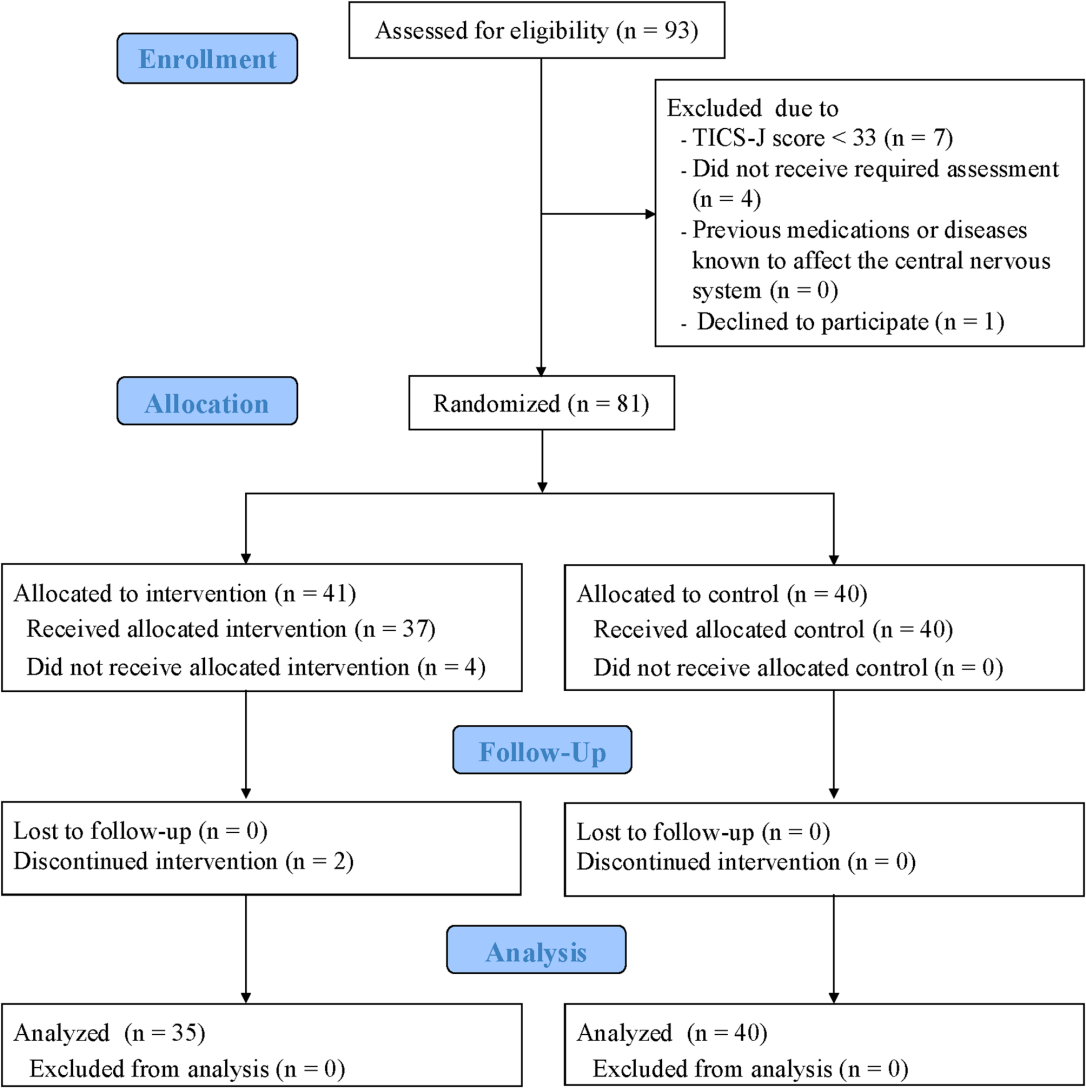
The outline of the study design and procedures

Figure 2 illustrates an outline of the study design and procedures. To recruit participants, we used a letter from the municipal government involved in our joint research as well as flyers posted on the city’s website and in local stores. These documents included a brief explanation of the study aim, period, content of the intervention, and eligibility criteria for participation. Mailing address information was sourced from the municipal government. Approximately 3,850 letters were sent to community dwellers aged 65 and older who had previously participated in questionnaire investigations by the municipal government. Potential participants interested in the trial contacted us through the phone number provided in the recruitment letter.

### Intervention

#### Intervention overview

The intervention program comprised weekly group conversations facilitated by the PICMOA mobile application. The participants engaged in the remote group conversation program using smartphones at their homes once a week for approximately one hour for a duration of 12 weeks.

The PICMOA application is a mobile-based platform developed to facilitate structured group conversations. Users can upload photos related to pre-defined conversation themes and participate in weekly group conversation sessions via the application. More information about the application is provided in the protocol [18].

Intervention period and frequency were based on findings from our previous RCT conducted in face-to-face settings and previous systematic reviews of RCTs targeting older adults and cognitive function. Additionally, the intervention required participants to take daily photographs during each session. Therefore, intervention period and frequency were determined in consideration of feasibility, adherence, and potential benefit.

Each weekly session consisted of two structured components: (1) pre-session photo preparation and (2) a group conversation session.

#### Pre-session photo preparation

Before each intervention session, participants were instructed to take as many photographs as possible during their daily activities that they felt were relevant to the upcoming intervention session’s conversation theme, such as objects, environments, or activities related to the topic (e.g. preferred items, local landmarks). Then, they were asked to upload two photographs to the intervention server utilizing the camera integrated within the PICMOA application. Each weekly session had a theme, totaling twelve themes adopted from previous trials [13, 14]. These themes were qualitatively designed through discussions among research team members to promote cognitive engagement and encourage activities during the photo preparation phase. A full list of the 12 conversation themes is provided in Supplementary File 1.

#### Group conversation session structure

Each session consisted of an opening greeting, a brief icebreaker talk, reminders about effective participation strategies (e.g., active listening, balanced speaking), PICMOA-based group conversation (i.e., the main group conversation), and the completion of a feedback form at the end. The session was facilitated by research staff and the PICMOA mobile application. The total duration of each session was approximately one hour. Each conversation group comprised four members, and ten groups were formed for the intervention. Group composition was arranged based on participants’ schedules and gender balance.

During the PICMOA-based group conversation in the intervention session, each participant in the designated group gave a one-minute speech about their two selected photographs taken and uploaded before each session, as described earlier.

Subsequently, other participants asked questions related to the photographs and the presenter responded to the questions in a natural conversational context, with a duration of two minutes for each photograph.

In one session, for example, the theme for the week was “favorite items.” Participants took photographs of their favorite objects, or ones they enjoyed, such as their favorite cup or musical instrument, and uploaded two photographs to the server. During the group conversation session, the system presented each photograph to the group members on their smartphones via the intervention application. The participant provided a one-minute explanation of the photo shown. After that, other group members asked questions related to the photo or story, and the presenter responded using natural conversational flow. This interaction was carried out for a total of eight photos, two for each participant, for a total of four group members. This format was repeated every week using a different theme. An automated voice prompt embedded in the PICMOA application guided the conversation flow by providing structured cues. In addition, research staff supported the session by controlling the turn order and ensuring balanced participation.

#### Device setup and training

The intervention group was provided with an Android smartphone (approximately 6.5 inches), while the control group was provided with a tablet PC (approximately 10 inches). Both devices were connected to the internet via SIM cards installed by the research team, ensuring that all participants could join sessions without needing their own internet access, as both the PICMOA application and zoom required an internet connection. After participants were informed of the random allocation result, they received the equipment and a manual. First, participants practiced basic device use and application individually with research staff. Second, we conducted the test sessions for each group to confirm that all members could connect to the conversation session. Throughout the training and intervention phases, participants received timely technical support through phone calls when they encountered difficulties operating the device or application.

#### Intervention schedule and group assignment

For operational purposes, the study period was divided into two periods (intervened June 2022 to August 2022 and October 2022 to December 2022). Each participant participated for 12 weeks concurrently without staggered timing, and all participants completed 12 intervention or control sessions. Both intervention and control sessions were provided at fixed times on the same day each week, as assigned to each group.

#### Control group

Participants in the control group watched weekly health education video sessions on successful aging for approximately one hour a week for 12 weeks. The videos were delivered via a webinar [26] on set days and times during the intervention period. Participants were invited to join the webinars live, for approximately one hour per session. Each session included confirming participants’ attendance, one-way greetings, watching the video, and completing a feedback form. The webinars were one-way, with no conversations allowed with the moderator or other participants. We expected that comparing the effects of the new intervention with those of traditional interventions, such as health education, would allow for a more accurate evaluation of the specific effects of the new intervention and control for the influence of using unfamiliar electronic devices on the intervention’s effects. The frequency, session duration, and intervention period in the health education control group were matched with the intervention program.

#### Technical support and session management

Research staff attended all intervention and control sessions via the application or Zoom, facilitating the sessions, providing timely technical support when needed, and monitoring attendance by confirming participants’ presence. If a session could not be conducted due to internet connection issues or other problems, it was rescheduled for a later date.

The members of each conversation group remained consistent across all intervention sessions to foster group dynamics and social connections. In the case of a withdrawal during the intervention period, research staff joined the group conversation as a substitute to ensure that the intervention’s intensity was maintained, since different numbers of group members can lead quantitative differences in the amount and duration of speaking, potentially impacting the effect sizes.

The feasibility of using a smartphone application for the PICMOA intervention was confirmed through a small-scale pilot test as part of an explanatory single-arm intervention study (UMIN000048149). Although formal feedback items were not included in the RCT evaluation, participant feedback was collected through timely technical support, preliminary testing, and post-evaluation.

### Outcomes

#### Main outcomes

We designated cognitive functioning as the primary outcome measure, especially with a focus on linguistic and executive function, since we hypothesized that PICMOA may enhance these fundamental functions in conversation. The standardized neuropsychiatric tests were administered by highly trained clinical psychologists over the telephone. The clinical psychologists coded and documented the participant’s responses and scores on the test form during testing. Data were entered based on the documents, and the first author checked for data errors.

To assess global cognitive functioning, the TICS-J [25] was employed, and the total score was calculated accordingly (ranging from 0 to 41). Verbal fluency tests, encompassing both semantic and phonemic fluency, were administered to assess linguistic function and memory retrieval abilities, which are believed to reflect executive control [27]. Semantic fluency was assessed using an animal-naming fluency test [28], in which participants were required to name animals, while the phonemic fluency test required them to produce as many words as possible, beginning with the phoneme “ka,” within one minute. We counted the total number of words generated (no fixed upper limit). To assess verbal working memory capacity, the Digit Span Forward and Backward tests from the Wechsler Adult Intelligence Scale – Fourth Edition (WAIS-IV) [29] were employed. The participants were asked to reproduce the numbers generated by the clinical psychologists in the exact order for the Digit Span test (ranging from 0 to 16), and in reverse order for the Backward test (ranging from 0 to 14).

#### Secondary outcomes

Selected indicators related to psychological and social well-being, assessed by questionnaires, were designated as a secondary outcome.

The Multidimensional Scale of Perceived Social Support (MSPSS) [30, 31] and the UCLA Loneliness Scale-10 item version [32] were selected to assess social support and loneliness, respectively. The MSPSS comprises 12 items and measures the perceived level of social support within a network. The total score was calculated by averaging the scores of each item (ranging from 0 to 7), with higher scores indicating greater social support. The UCLA Loneliness Scale-10 item version was used to measure loneliness. This scale is a short form derived from the 20-item version to reduce respondent burden [32]. The total score was calculated by summing each score (ranging from 10 to 40), with higher scores reflecting a greater degree of loneliness.

The Japanese version of the Geriatric Depression Scale-short form (GDS-15) [33], the 5-item World Health Organization Well-Being Index (WHO-5) [34], and the Health Utilities Index Mark 3 (HUI3) [35] were employed to evaluate depressive symptoms, psychological well-being, and health-related quality of life (HRQOL), respectively. The GDS-15 is a 15-item and screens the depression (ranging from 0 to 15), with higher scores reflecting more depressive symptoms. The WHO-5 was used to evaluate psychological well-being. The percentage score of WHO-5 was calculated (ranging from 0 to 100), with higher scores reflecting better well-being. The HUI3 measured HRQOL. The multi-attribute utility score was calculated using the formula (ranging from − 0.36 to 1.00), with higher scores indicating better HRQOL.

#### Covariates

Age, gender, education, and familiarity with smartphones, which was collected by questionnaire, were used as covariates. Age was treated as a continuous variable. Education assessed the years of education and was dichotomized into “<13 years,” and “≥13 years.” Familiarity with smartphones was measured by asking how often they use smartphones. The question had four response options: “usually,” “sometimes,” “rarely,” and “never.” For analysis, we dichotomized the responses into “frequent use” (“usually”): 1; and “sometimes/rarely/never” (“sometimes,” “rarely,” or “never”): 0.

### Sample size

G*power [36] was used to calculate sample size under the following conditions: two-sided hypothesis test; small to medium effect size (f = 0.175); 80% power, an analysis of variance model between groups over time; two measurements for principle outcomes, with a correlation of 0.5 among repeated measures; and a 5% alpha level. Accordingly, 80 participants were required for this trial, considering a 15% dropout rate. These conditions were determined based on the small effect size observed in a previous face-to-face trial [13], prior studies on face-to-face social interventions that anticipated medium effect sizes [37], and correlations among repeated measures derived from data in earlier trials [13, 14].

### Statistical analysis

Linear mixed models with random intercepts for outcome measurements were performed to estimate the impact of the PICMOA intervention. The model included the groups assignment factor (intervention: 1, control: 0), time factor (pre: 0, post: 1), and their interaction term (time × group), which was interpreted as the intervention effect. This study used a repeated-measures design. Ordinary least-squares regression is not appropriate because it violates the assumption of independence. By contrast, a linear mixed model with random intercepts explicitly models individual differences at baseline as random effects, improving the accuracy of estimates by accounting for within-subject correlations. Different from repeated-measures analysis of variance, linear mixed models separate variability sources (random intercepts and residuals), offering more precise and interpretable results.

Considering that familiarity with smartphones at baseline may have affected the effectiveness of interventions, we examined whether the intervention effects varied with the levels of smartphone familiarity (frequent use: 1, sometimes/rarely/never: 0) based on a priori hypothesis. Since age, gender, and education may be confounding factors associated with both cognitive function and smartphone familiarity, we controlled these variables in the ancillary analyses.

All analyses were conducted using the software R, and linear mixed models were implemented with the lmer function from the lme4 package [38]. A statistical significance threshold of *P* < 0.05 was applied.

## Results

### Participant flow and recruitment

Participants flow is illustrated in the CONSORT diagram (Fig. 1). Recruitment began in March 2022. From April to September 2022, 93 participants were screened for eligibility and 81 participants were randomly assigned into groups (intervention: *n* = 41; control: *n* = 40). All participants in the control group were included in the final analysis, whereas four participants declined consent after randomization (health related reasons: *n* = 3; perceived incompatibility with intervention: *n* = 1) and two participants discontinued the intervention in the intervention group (Internet access problems: *n* = 1; perceived incompatibility with the intervention: *n* = 1). Finally, 75 participants (intervention: *n* = 35; control: *n* = 40) who completed the intervention and pre-post evaluations were analyzed. There were no negative or unintended effects in either group. Table 1 presents the baseline characteristics.

**Table 1:** Baseline characteristics

Characteristics	Intervention, *N* = 35	Control, *N* = 40
Age	73.23 (4.61)	73.60 (4.99)
Gender		
Female	22 (63%)	24 (60%)
Male	13 (37%)	16 (40%)
Education		
< 13 years	17 (49%)	19 (48%)
≥ 13 years	18 (51%)	21 (53%)
Smartphone familiarity		
Frequent use	21 (60%)	30 (75%)
Sometimes/rarely/never	14 (40%)	10 (25%)
Living status		
Living alone	8 (23%)	9 (23%)
Living with family members	27 (77%)	31 (78%)
Subjective economic status		
Very comfortable	1 (3%)	3 (8%)
Somewhat comfortable	9 (26%)	12 (30%)
Fair	18 (51%)	21 (53%)
Somewhat difficult	7 (20%)	4 (10%)
Very difficult	0 (0%)	0 (0%)
Disease status		
Hypertension	11 (31%)	9 (23%)
Heart disease	1 (3%)	4 (10%)
Diabetes	5 (14%)	5 (13%)
Cancer	1 (3%)	4 (10%)
TICS	36.14 (1.48)	36.05 (1.75)
Letter fluency	13.23 (3.80)	12.85 (3.48)
Categorical fluency	17.14 (3.60)	16.25 (4.25)
Digit span forward	9.11 (2.00)	9.00 (1.74)
Digit span backward	8.06 (1.98)	8.10 (2.00)
UCLA loneliness	19.83 (5.72)	17.58 (4.51)
Social support	5.10 (1.09)	5.75 (0.88)
WHO5	67.54 (16.34)	76.70 (11.81)
HUI3	0.50 (0.35)	0.59 (0.31)
GDS15	2.77 (2.71)	2.08 (1.90)

Mean, (SD); n (%)

### Intervention effects on outcome measurements

Table 2 demonstrates the intervention effects on cognitive function and the selected indicators of psychological and social well-being, comparing the pre- and post-intervention scores between the intervention and control groups. Table 2 presents the intervention effects on cognitive function. No significant group × time interactions were found in other cognitive outcomes, including TICS-J (β = −0.74, 95% CI = − 1.60 to 0.13, SE = 0.44, *p* = 0.10), letter fluency (β = −0.02, 95% CI = − 1.45 to 1.40, SE = 0.73, *p* = 0.97), categorical fluency (β = −0.96, 95% CI = −3.27 to 1.35, SE = 1.18, *p* = 0.42), digit span forward (β = −0.74, 95% CI = − 1.49 to 0.02, SE = 0.38, *p* = 0.06), and digit span backward (β = 0.03, 95% CI = − 1.01 to 1.07, SE = 0.53, *p* = 0.96).

Similarly, no significant group × time interactions were observed for UCLA Loneliness (β = −1.43, 95% CI = − 2.92 to 0.07, SE = 0.76, *p* = 0.07), social support (β = 0.10, 95% CI = − 0.18 to 0.37, SE = 0.14, *p* = 0.50), WHO-5 (β = 3.76, 95% CI = − 1.78 to 9.30, SE = 2.83, *p* = 0.19), HUI3 (β = −0.03, 95% CI = − 0.16 to 0.09, SE = 0.06, *p* = 0.61), or depressive symptoms (β = −0.14, 95% CI = − 0.80 to 0.53, SE = 0.34, *p* = 0.69).

**Table 2:** The intervention effects on cognitive and psychological outcomes

	Pre		Post		Estimate								
	Intervention	Control	Intervention	Control	Time			Group			Time × Group		
	Mean (SD)		Mean (SD)		Coefficients (95% CI)	SE	*P*	Coefficients (95% CI)	SE	*P*	Coefficients (95% CI)	SE	*P*
TICS	36.14 (1.48)	36.05 (1.75)	36.06 (2.31)	36.70 (1.84)	0.65 (0.06, 1.24)	0.30	0.03	0.09 (−0.75, 0.93)	0.43	0.83	−0.74 (−1.60, 0.13)	0.44	0.10
Letter fluency	13.23 (3.80)	12.85 (3.48)	13.43 (4.45)	13.08 (3.74)	0.23 (−0.75, 1.20)	0.50	0.65	0.38 (−1.37, 2.12)	0.89	0.67	−0.02 (−1.45, 1.40)	0.73	0.97
Categorical fluency	17.14 (3.60)	16.25 (4.25)	16.83 (4.00)	16.90 (4.83)	0.65 (−0.93, 2.23)	0.81	0.42	0.89 (1.01, 2.79)	0.98	0.36	−0.96 (−3.27, 1.35)	1.18	0.42
Digit span forward	9.11 (2.00)	9.00 (1.74)	8.63 (1.75)	9.25 (1.79)	0.25 (−0.26, 0.76)	0.26	0.34	0.11 (−0.71, 0.94)	0.42	0.79	−0.74 (−1.49, 0.02)	0.38	0.06
Digit span backward	8.06 (1.98)	8.10 (2.00)	8.49 (2.19)	8.50 (2.29)	0.40 (−0.31, 1.11)	0.36	0.27	−0.04 (−0.10, 0.91)	0.49	0.93	0.03 (−1.01, 1.07)	0.53	0.96
UCLA loneliness	19.83 (5.72)	17.58 (4.51)	18.83 (5.02)	18.00 (4.28)	0.42 (−0.60, 1.45)	0.52	0.42	2.25 (0.05, 4.46)	1.13	0.05	−1.43 (−2.92, 0.07)	0.76	0.07
Social support	5.10 (1.09)	5.75 (0.88)	5.15 (1.21)	5.70 (0.98)	−0.05 (−0.23, 0.14)	0.10	0.64	−0.65 (−1.12, −0.18)	0.24	< 0.01	0.10 (−0.18, 0.37)	0.14	0.50
WHO5	67.54 (16.34)	76.70 (11.81)	68.00 (14.74)	73.40 (11.65)	−3.30 (−7.08, 0.48)	1.93	0.09	−9.16 (−15.32, −2.99)	3.16	< 0.01	3.76 (−1.78, 9.30)	2.83	0.19
HUI3	0.50 (0.35)	0.59 (0.31)	0.53 (0.31)	0.64 (0.24)	0.06 (−0.03, 0.14)	0.04	0.19	−0.08 (−0.22, 0.05)	0.07	0.23	−0.03 (−0.16, 0.09)	0.06	0.61
GDS15	2.77 (2.71)	2.08 (1.90)	2.69 (2.63)	2.13 (2.37)	0.05 (−0.40, 0.50)	0.23	0.83	0.70 (−0.39, 1.78)	0.56	0.21	−0.14 (−0.80, 0.53)	0.34	0.69

### Intervention effects differences by familiarity with smartphone use at baseline

**Fig. 3:**
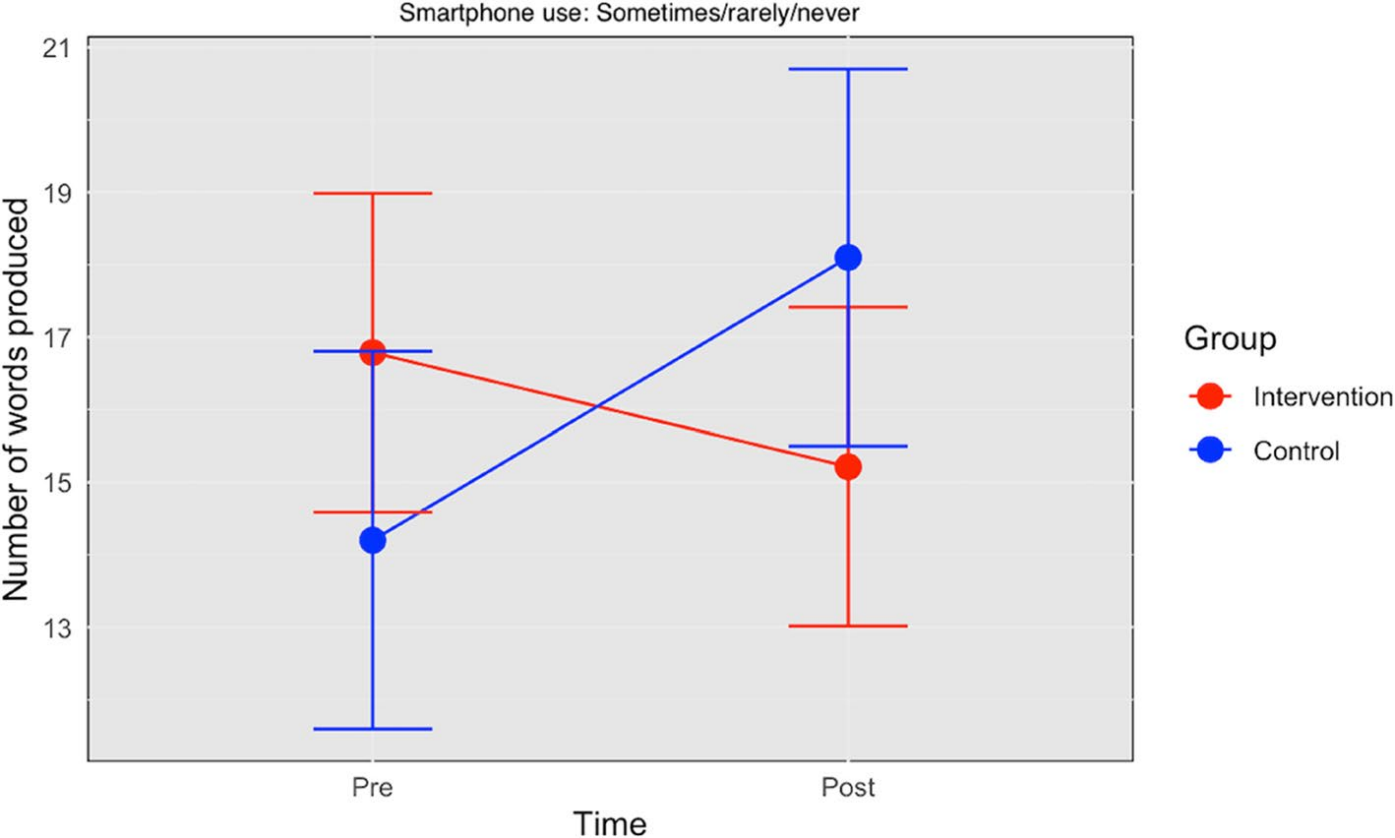
The intervention effects on categorical fluency among those unfamiliar with smartphones at baseline. Note Error bars represent ± 1 standard error of the model-estimated marginal means

Three-way interactions between group, time, and smartphone use were confirmed in the ancillary analyses (Supplementary Table 2). Figure 3 depicts the intervention effects based on smartphone use at baseline. The intervention effect was different by familiarity with smartphone use at baseline (Group × Time × Smartphone familiarity: b = 6.43, 95% CI = 1.66 to 11.20, SE = 2.47, *P* = 0.01). There were significant negative effects of intervention on categorical fluency in those who were not familiar with smartphone use before the intervention even after controlling for age, gender, and education (Time × Group: b = −5.47, 95% CI = −9.40 to 1.54, SE = 2.04, *P* = 0.009). Conversely, no significant positive effects of the intervention on categorical fluency in those who were familiar with smartphone use at baseline were observed with a net effect of b = 0.96 (−5.47 + 6.43). No statistically significant three-way interactions with smartphone familiarity were observed for other outcomes.

## Discussion

### Principal findings and interpretation

This trial evaluated the effect of PICMOA, an application-based remote group conversation intervention, on cognitive function and selected indicators of psychological and social well-being based on RCT. Overall, the results did not show a significant improvement in cognitive function and psychological metrics during the intervention period. However, there were significant negative effects of intervention for those who were not familiar with smartphones before intervention.

Despite several beneficial effects of conversational intervention on the verbal fluency test reported in previous studies, the PICMOA trial did not show overall significant improvements in cognitive function and psychological metrics. A previous RCT with 30-minute weekly face-to-face sessions of PICMOR for 12 weeks demonstrated a significant improvement in phonemic fluency, compared to the control group with free group chat [13]. Although conversation intervention studies on cognitive function remain limited and exploratory, Dodge et al. reported beneficial effects of a web-based conversational intervention over 6 weeks [39] and 6 months [40] of administration on cognitive outcomes. Differences between the results of PICMOA trial and those of previous RCTs can be explained as follows:

First, the intensity or duration of the intervention might not be sufficient, especially in the remote format. Although the intervention was delivered with the same intensity and duration as the face-to-face intervention that showed significant improvements [13], this remote intervention did not show any significant benefits. One possible reason could be that conditions specific to the home-based digital intervention, including the participant’s home environment and remote interaction with other participants and staff, may have reduced the strength of social interaction as a stimulus and weakened concentration and motivation for intervention.

This partially aligns with a recent meta-analysis based on previous RCTs that reported effect modifiers for computerized cognitive training intervention and suggested that home-based delivery was not effective [41]. The lack of significant effects in this study might reflect similar challenges in maintaining engagement and cognitive stimulation in remote settings. Specifically in remote formats, the lack of physical co-presence may reduce the richness of social cues, reciprocal responsiveness, and immediacy that are key factors driving cognitive engagement in conversational interventions. These factors suggest that intervention protocols developed for face-to-face settings may not be directly transferable to remote settings without adaptation. Future intervention designs may need to consider whether increased session frequency, extended duration, or additional strategies to increase engagement are necessary to achieve similar effects in remote delivery.

A comprehensive understanding of the effect of an application-based remote social intervention on cognitive stimulation is lacking. A previous study suggested that remote verbal conversation with equipment was more difficult than in-person conversation, as an experiment demonstrated that remote verbal interaction had an overall slower rate than in-person conversation [42]. Further, we experienced interruptions during the intervention session due to occasional poor internet connection. These specific instrumental and environmental issues may have had some impact on intervention efficacy.

According to Mehrabian [43], voice, tone, and non-verbal communication also play an important role in conversation. The brain processes not only linguistic information but also multifaceted non-linguistic information, including voices [44], facial expressions [45], and emotional state [46] of other speakers while communicating. These underlying specific processes may contribute to the maintenance of an individual’s cognitive ability. However, the amount of this non-linguistic information necessary to stimulate brain activity could be reduced/lost through the screen of a small smartphone. Although the PICMOA is designed to display real-time videos of each of the four participants’ faces at the bottom of the screen during the session, the smartphones (6.5-inch screen display) may not be large enough to capture information on the social dynamics of the group. Additionally, the small display could hinder usability and visibility, as well as influence the operation performance for older adults [47]. Further studies are warranted to understand the knowledge gap on whether instrumental conditions, such as screen size, influence the stimulus strength of remote social interaction, and whether in-person and application-based remote social interventions have different effect sizes.

Second, the short training period, or the 12-week intervention duration, also may not be sufficient to accustom older adults to new equipment/applications for them to benefit from the intervention. Particularly, although we developed the application with a user-friendly interface and provided opportunities for participants to practice operating the smartphone, there was still a possibility that unfamiliarity with the equipment would reduce intervention performance and weaken its effectiveness. A previous study that examined the effect of behavioral intervention based on smartphone application on preoperative anxiety in pediatric patients reported that the group with previous experience using smartphone applications had a significantly higher response to the intervention compared with the group with no experience [48]. A previous experiment [19] also reported that individuals who were unfamiliar with the digital equipment at baseline produced fewer utterances during a one-on-one conversational intervention with a robot, which may reflect the parameter of output in conversation. The effect of familiarity with smartphone use on the effectiveness of eHealth interventions is not yet clear. Further investigation is needed on whether a longer period of intervention or sufficient training period for a population unfamiliar with digital devices enhance the digital intervention effect among older adults.

Notably, this study observed different patterns in the intervention effects on category fluency by their smartphone familiarity at baseline; the significant negative effect was observed for those who were unfamiliar with smartphones at baseline and no significant effect was observed for those who were familiar with smartphones at baseline. The negative effect of PICMOA among individuals who were unfamiliar with smartphones is partially consistent with a previous in-person RCT in terms of the effect on verbal fluency, as the study consistently confirmed the negative effect on categorical fluency among older adults’ with higher levels of NfL, implying a relatively damaged neuronal state [14]. The category fluency test is a neuropsychological assessment widely used to evaluate executive function and lexical access [49] which closely links to conversation. Although no conclusions on the overall effect can be drawn, consistent results on verbal fluency outcomes may reflect negative effects of conversation intervention on specific cognitive functions under certain conditions, including executive function and lexical access. One direction for future research is to appropriately personalize interventions so that they can positively impact people in various states.

### Feasibility and implementation considerations

This study provides practical insights into the feasibility and limitations of scalable, digitally inclusive prevention approaches, which may have implications for future discussions on care delivery models in ageing societies. With regard to implementation, the remote delivery of the intervention has several practical advantages. Compared with center-based programs, there may be fewer overhead costs associated with staffing and facility rental. Although this study provided participants with smartphones with pre-installed mobile networks to ensure uniform conditions, participants in real-world implementation could use their own smartphones and internet access, resulting in lower up-front costs. However, the use of smartphones and tablet devices is hindered by the digital literacy of older adults and a lack of consideration for their motor function and sensory impairments [50]. While such challenges are manageable in a controlled research environment to a certain degree, large-scale implementation would require a structured technical support system and optimized environments to accommodate digital competency levels, as well as physical and sensory limitations.

### Strengths

This study has the following strengths. First, the PICMOA adds to evidence on digital technology interventions through a strong RCT design. Notably, this study was a digital intervention conducted under COVID-19 pandemic-related restrictions in Japan, providing practical knowledge and directions for future research on digital technology interventions during public health crises. Although the primary findings did not reach statistical significance, they provide valuable insight into the boundaries and contextual factors that may limit the effectiveness of digital health interventions in older adult populations. Based on the study findings, it is necessary to conduct future studies to determine: (1) whether a longer period of intervention or sufficient training period for a population unfamiliar with digital devices reduces the negative effects of unfamiliarity and enhances the feasibility and continuity of application-based interventions for older adults, (2) the influence of instrumental conditions (such as screen size) on digital technology intervention, and (3) the intensity of stimulus of an in-person intervention compared to an application-based remote social intervention.

Lastly, the participants in this trial included older adults with subjective cognitive concern assessed by the KCL, which is the target population for preventive care in Japan’s public policy. Therefore, focusing on this population holds significant clinical and policy relevance.

### Limitations

This study has several limitations. First, the duration or intensity of the intervention may not be sufficient. The relatively short duration might have limited the potential for cumulative effects, particularly in a remote delivery setting. Second, the participants were older adults who voluntarily participated in this study and were recruited from only one local region. Therefore, selection bias may have occurred, and careful discussion is required for generalizability. Third, the intervention group was provided with a standardized smartphone to ensure consistency of the intervention environment. However, not using personal smartphones may have strengthened the influence of unfamiliarity with smartphones on the results. It may be necessary to test whether using one’s own smartphones and strengthening pre-training modify the intervention results. Fourth, baseline TICS-J scores were relatively high and narrowly distributed. Although this did not indicate a clear ceiling effect, the limited range of variation may have limited the potential for noticeable improvement, especially during the relatively short intervention period.

Fifth, this study had six consent withdrawals in the intervention group. Although the randomized method was used to allocate participants to the intervention or control group without bias, the potential biases in the intervention group likely affected intervention continuation. Lastly, this trial could not address the potential interaction effects with other neurological markers utilizing MRI and biomarkers (e.g., NfL) to reduce contact with the participants who were older and at risk of contracting COVID-19. Since the beneficial effects of PICMOR have been reported in individuals with low NfL levels [14], neurological markers might have similarly interacted with this PICMOA intervention. To address these limitations, another trial has been launched implementing a six-month intervention introducing a longer duration, an updated version of the intervention platform, and the evaluation of blood-based biomarkers [51]. This extended study is expected to provide further insights into the potential long-term benefits of the application-based conversation program. Furthermore, this study conducted no qualitative analysis of participants’ perceptions of the cultural or social appropriateness of the themes of conversation, users’ experience of the intervention, or process data such as the number of photos taken, levels of engagement during the group conversation, or changes in group dynamics. A mixed-methods approach in future studies could potentially reveal contextual factors that influence participants’ perceptions and engagement. Further exploratory analyses are necessary to address these important aspects in future research.

## Conclusions

This trial evaluated the effects of PICMOA, an application-based remote group conversation intervention, on the cognitive function and psychological and social well-being (based on selected indicators) of older adults. To conclude, the 12-week PICMOA intervention did not result in overall significant improvements in cognitive and psychological outcomes during the intervention period. However, this study provides directions for future research on digital technology interventions. These include investigating whether a longer intervention or more extensive training period could enhance the effects of application-based interventions for older adults, understanding the effect of instrumental conditions on digital interventions, and examining the differences in stimulus intensity between in-person and remote application-based social interventions. Based on the results, further studies are warranted to better understand the knowledge gaps.

## Supplementary Information

Supplementary Material 1.

Supplementary Material 2.

## Data Availability

The datasets collected in this study are available upon reasonable request from the corresponding author.

## Abbreviations

RCT: Randomized controlled trial
MCI: Mild cognitive impairment
PICMOR: Photo-Integrated Conversation Moderated by Robots
PICMOA: Photo-Integrated Conversation Moderated by Application
UMIN: University Hospital Medical Information Network
TICS-J: Telephone Interview for Cognitive Status in Japanese
KCL: Kihon Checklist
WAIS-IV: Wechsler Adult Intelligence Scale – Fourth Edition
MSPSS: Multidimensional Scale of Perceived Social Support
GDS-15: Geriatric Depression Scale-short form
WHO5: 5-item World Health Organization Well-Being Index
HUI3: Health Utilities Index Mark 3
HRQOL: Health-related quality of life
NfL: Neurofilament light chain
