# Supplementary material for: A 12-week application-based group conversation intervention on cognitive health and psychosocial well-being among older adults during the COVID-19 pandemic: a randomized controlled trial
Source: BMC Geriatr. 2025 Oct 14;25:774. doi: 10.1186/s12877-025-06444-0 (PMC12523125; doi:10.1186/s12877-025-06444-0)
Supplement: Supplementary file 1 — Supplementary Material 1. [file 12877_2025_6444_MOESM1_ESM.docx]

Supplementary Table 1. List of the 12 themes in the PICMOA intervention

| 1 | Favorite thing |
| --- | --- |
| 2 | Neighborhood landmarks |
| 3 | Feeling the season |
| 4 | Favorite foods |
| 5 | For my health |
| 6 | Tips for daily living |
| 7 | Being mindful or disaster prevention |
| 8 | Funny storied and mistakes |
| 9 | Things to get rid of |
| 10 | Found on a 10 minutes walk |
| 11 | Cleaning, before and after |
| 12 | Starting something new |
