# Supplementary material for: A 12-week application-based group conversation intervention on cognitive health and psychosocial well-being among older adults during the COVID-19 pandemic: a randomized controlled trial
Source: BMC Geriatr. 2025 Oct 14;25:774. doi: 10.1186/s12877-025-06444-0 (PMC12523125; doi:10.1186/s12877-025-06444-0)
Supplement: Supplementary file 2 — Supplementary Material 2. [file 12877_2025_6444_MOESM2_ESM.docx]

Supplementary Table 2. Three-way interactions among group, time, and baseline smartphone use

| Item | Estimate | Standard error | P |
| --- | --- | --- | --- |
| (Intercept) | 16.87 | 6.46 | 0.01 |
| Age | -0.03 | 0.08 | 0.69 |
| Gender | 0.05 | 0.83 | 0.95 |
| Education | 0.66 | 0.81 | 0.42 |
| Intervention | 2.63 | 1.76 | 0.14 |
| Time | 3.90 | 1.56 | 0.01 |
| Smartphone familiarity | 2.88 | 1.57 | 0.07 |
| Intervention * Time | -5.47 | 2.04 | 0.01 |
| Intervention * Smartphone familiarity | -2.20 | 2.15 | 0.31 |
| Time * Smartphone familiarity | 4.33 | 1.80 | 0.02 |
| Intervention * Time* Smartphone familiarity | 6.43 | 2.47 | 0.01 |
